# Fetal radiation doses and subsequent risks from X-ray examinations: Should we be concerned?

**Published:** 2013-11

**Authors:** Ali Chaparian, Mahdi Aghabagheri

**Affiliations:** 1 *Department of Medical Physic, Shahid Sadoughi University of Medical Sciences, Yazd, Iran.*; 2 *Department of English Language, Shahid Sadoughi University of Medical Sciences, Yazd, Iran.*

**Keywords:** *Pregnant women*, *Radiography*, *Radiation Effects*, *Fetus*, *Monte Carlo method*

## Abstract

**Background:** Pregnant women are sometimes exposed to ionizing radiation in radiology examinations for various reasons. In such cases, the radiation dose to the conceptus and subsequent risks should be estimated.

**Objective: **The purpose of this study was the calculation and presentation of fetal dose and subsequent risks resulted from different X-ray examinations.

**Materials and Methods:** An analytical simulation study was conducted and six common radiographies in different views and three types of special examinations were evaluated. The entrance skin exposure (ESE) was measured using a solid-state dosimeter. A Monte Carlo program was used in order to simulate different views of X-ray examinations and calculate the radiation doses received by the conceptus for every view of each examination. Then the risk of childhood cancer and small head size were calculated for different radiographies.

**Results: **The fetal doses and consequence risks of the small head size and childhood cancer for the radiographs of chest, skull, and sinuses were negligible but the risks of childhood cancer and small head size due to radiographies of abdomen, lumbar spine, and pelvis areas were ponderable.

**Conclusion:** Results of this study can be used for the pregnant women radiographies management.

## Introduction

In some cases, it is possible that a female patient would not aware of her pregnancy in the radiology examination time, or it is probable that she would be aware of her pregnancy but owing to the emergency issues or some accidents, radiology examinations seem inventible. In these two cases, the patient would be worried due to the radiation danger for the conceptus, and the patient would be hesitant in the case of pregnancy termination. Also, this concern can cause a real challenge for the physicians or radiologists. 

In these cases, received doses by conceptus should be estimated. The awareness in the case of received dose by conceptus can be helpful to assess the benefits versus radiation risks and it can lead to design some examinations with the lower doses. In addition, aware physicians can better guide his patient in the case of potential perils of radiation. The data of previous studies focused on the animal and human subjects considering potential radiobiological effects on conceptus. The primary sources of human data mostly included the survivors of the Hiroshima and Nagasaki atomic bomb. In those accidents, a group consisting approximately 2800 pregnant women exposed to radiation, 500 of whom received a conceptus dose of more than 10 mGy (1). 

The probable impacts of radiation on a conceptus consist of prenatal death, intrauterine growth limitation, small head size, strict mental retardation, reduced IQ (intelligence quotient), organ malformation, and childhood cancer. These impacts are relevant to the radiation dose to the conceptus and the phase of conceptus growth at which the exposure takes place. The mentioned issues are summarized in table I (2). Different factors affecting fetal dose during radiological examinations were presented by Karam. These factors included X-ray tube voltage (kVp), tube current (mA), total filtration (mm Al), exposure time (seconds), the tube to the patient distance, the numbers and location of exposures (3).

Some guidelines on radiation protection of pregnant patients at the time of radiology tests were presented by The American College of Radiology. The guidelines objectives were to assist practitioners to identify pregnant patients, to prevent unnecessary radiation of pregnant women, to optimize examinations for effective radiation dose management, and to develop some strategies to determine and evaluate the potential effects of radiation delivered to pregnant patients (4). 

A review study was conducted by Linet *et al* in 2008. The current and historical expected doses for common X-ray examinations as well as the epidemiologic review on the significance of maternal prenatal, children’s postnatal and parental preconception X-ray examination on successive risk of childhood malignancies were summarized. Cancer risk estimates were related to some factors such as trimester and radiological examination type, and the number of taken films. It was also discussed the methods limitations employed in epidemiologic studies to assess pediatric cancer risks (5).

The purpose of this study was the calculation and presentation of fetal dose and subsequent risks resulted from different views of the common radiology and some special examinations. This procedure was conducted by using a Monte Carlo simulation program, some measurements, and calculations.

**Table I:** Fetal effects from low-level radiation exposure

**Absolute Incidence ** *		**Threshold dose at which an effect was observed (mGy)**	**Most sensitive period** **after conception (day)**	**Effect**
ND	No data		0-8	Prenatal death
NO DATA	200		8-56	Growth retardation
NO DATA	250		14-56	Organ malformation†
0.05-0.10%	No threshold observed		14-105	Small head size
0.04% ‡	100		56-105	Severe mental retardation
NO DATA	100		56-105	Reduction of IQ (intelligence quotient)
0.017% §	No threshold observed		0-77 (first trimester)	Childhood cancer

* Absolute incidence is defined as the percentage of exposed fetuses in which an effect is expected to be observed with a dose of 1 mGy.

† Organ malformation is defined as malformation of an organ outside the central nervous system. Data regarding the most sensitive period after conception are from animal studies.

‡ An absolute incidence of 0.02% also was observed after radiation exposure of more than 500 mGy at 112-175 days after conception.

§ The baseline risk for unexposed fetuses is 1 in 1500 or 0.067%. An absolute incidence of 0.0043% per milligray was observed for fetuses with radiation exposure in the second and third trimesters.

## Materials and methods

**X-ray examinations**

An analytical simulation study was performed and six common radiographies in different views and three types of special examinations (a kind of radiography applying the contrast media) were investigated. The common radiographic examinations included: radiography of the skull [in three views of anterior-posterior (AP), posterior-anterior (PA) and lateral (Lat)], chest [in two views of (PA) and (Lat)], lumbar spine [in seven views of (AP), (PA), (Lat), right anterior-posterior oblique (RAO), left anterior-posterior oblique (LAO), right posterior-anterior oblique (RPO) and left posterior-anterior oblique (LPO)], abdomen [in two views of (AP) and (PA)], pelvis [in two views of (AP) and (PA)], and paranasal sinuses (Waters view). The special examinations included: intravenous pyelography (IVP), cystogrphy and oral cholecystography (OCG). 

The required radiographs for performing of IVP include: an X-ray of the abdomen before the injection, three X-rays of the kidneys: immediately, 5 minutes after the injection, and after the compression; an X-ray of total urinary tract: 15 minutes after the injection; finally, two x-rays of the bladder in full and empty situations. However, supplementary radiographs may be required in cases such as lack of renal excretion. The necessary radiographies of Cystogrphy include an AP view of the bladder before the injection of contrast material and views of AP, Lat, LPO and RPO after the injection. 

Sometimes complementary radiographies such as PA and axial views are also needed in special cases. The required radiographs for performing of OCG include views of LAO (the patient sleeps in a prone situation then the right portion of body is turned 20 to 30 degrees), RPO and sometimes AP (the patient sleeps in a right-lateral situation and beam is horizontally projected).

**Measurement of the entrance skin exposure (ESE)**

The entrance skin exposure (ESE) was measured using a solid-state dosimeter (Model 6001 UNFORS). Calibration of the dosimeter was done by Iran Secondary Standard Dosimeter Laboratory (SSDL) (Nuclear Research Center of Karaj) and the calibration factors for the range of X-ray energies used in this study were obtained. For measurement of ESE, the dosimeter was placed in the source to skin distance (SSD) associated to every view without the patient presence. Exposure factors [X-ray tube voltage (kVp), tube current (mA), exposure time (s)] related to every view was set on the X-ray units, and then measurements were performed. 

These measurements were repeated for seven X-ray machines available in the radiology departments of hospitals in Yazd. The appropriate exposure factors for performing the mentioned radiographies of an ordinary adult woman were determined for each machine by asking the expert technologists employed in every department.

**Calculation of fetal dose and subsequent risks**

The PCXMC (PC-based Monte Carlo program) (version 2.0) (6) developed by STUK (Radiation and Nuclear Safety Authority in Finland), was used in order to simulate different views of X-ray examinations. The dose calculation method in PCXMC was based on the Monte Carlo simulation. The Monte Carlo calculation of photon transport was according to stochastic mathematical simulation of interactions between photons and matter. 

The program calculated organ doses for a large number of organs/tissues of the patient by using anatomical data from mathematical phantom models. The anatomical data were based on the mathematical phantom models of Cristy and Eckerman (7). The program allowed a free choice of the x-ray examination techniques. The required input data for the simulation program included: definition of all views (location and size of the radiation field and projection angle) and radiation factors (ESE, kVp and total filtration) relating to different X-ray examinations. Performance and simulation of the different X-ray examinations (definition of and numbers of views) were based on standard guidelines e.g. Merrill’s atlas of radiographic positioning and procedures (8). 

After entering the above data into the program, the radiation doses received by the organs were calculated for every view of each examination. Then the absorbed dose of the uterus was used to represent the fetal dose before gestational week 12. According to table I, the risk of childhood cancer in this study was conservatively assumed to be 0.017% per mGy of fetal dose and small head size was also considered to be 0.10% per mGy. Thus, the risk of childhood cancer and small head size were calculated for different radiographs in the current study.

## Results

Table II shows fetal dose values and radiation induced risks of small head size and childhood cancer (per million) for different views related to the common X-ray examinations. The conceptus was received the highest dose in RPO and LPO views of lumbar spine, AP views of pelvis, abdomen, and lumbar spine; contrary to the mentioned fact, the lowest dose received in radiographies of the chest, skull, and sinuses. The conceptus dose was obtained for common view of the abdomen radiography, AP, and also in the unusual view, PA. Fetal dose for chest radiography was only calculated in (PA) and left lateral views since these views are recommended for better assessment of heart and lungs. 

The conceptus dose was also calculated for different views of the lumbar spine radiography. The AP view is common for the front one of this radiography, but as shown in table II, the conceptus dose was considerably decreased in PA comparing with AP view. This decrease also occurred in anterior oblique views of the lumbar spine in comparison with posterior views. Table III also demonstrates fetal dose values and radiation induced risks of small head size and childhood cancer (per million) for some special examinations distinguishing required types and views number. The conceptus was received the highest dose in Cystogram examination (5.81 mGy); contrary to the mentioned fact, the lowest dose received in OCG examination (0.55 mGy). 

The type and number of views for each examination were according to standard guidelines; however, fetal dose was separately shown for each view, such display is useful for calculating of fetal dose in cases that the complementary views are required for each examination. In IVP, for example, sometimes due to the secretion lack of kidneys, it is necessary to perform additional views or in accordance with the radiologists’ opinion, the type or number of views may be changed. For instance, instead of a local view of kidneys, the abdomen entire view could be taken. In such cases, the fetal dose can be calculated for additional views using data of table III.

**Table II:** Fetal dose values and radiation induced risks of small head size and childhood cancer (per million) for different views related to the common X-ray examinations

**X-Ray examination**	**View** *	**Uterus dose** **(mGy)**	**Risk of small head size** **(per million)**	**Risk of childhood cancer** **(per million)**
Abdomen				
	AP	1.217	1217	207
	PA	0.598	598	102
Chest				
	PA	0.000	0	0
	Lat	0.001	1	0
Pelvis				
	AP	1.226	1226	208
	PA	0.599	599	102
Skull				
	AP	0.000	0	0
	Lat	0.000	0	0
Lumbar Spine				
	AP	1.024	1024	174
	PA	0.569	569	97
	Lat	0.603	603	103
	RPO	1.490	1490	253
	LAO	0.827	827	141
	LPO	1.467	1467	249
	RAO	0.828	828	141
Sinuses	PA	0.000	0	0

* AP: anterior-posterior, PA: posterior-anterior, Lat: lateral, RPO: right posterior-anterior oblique, LAO: left anterior-posterior oblique, LPO: left posterior-anterior oblique, and RAO: right anterior-posterior oblique.

**Table III:** Fetal dose values and radiation induced risks of small head size and childhood cancer (per million) for different views related to three types of special examinations

**X-Ray examination** †	**View** *	**Number of view**	**Uterus dose for each view (mGy)**	**Total uterus dose (mGy)**	**Risk of small head size (per million)**	**Risk of childhood cancer (per million)**
IVP				4.720	4720	802
	Abdomen (AP)	2	1.218			
	Kidney (AP)	3	0.060			
	Bladder (AP)	2	1.052			
Cystography				5.805	5805	987
	AP	2	1.052			
	LPO	1	1.466			
	RPO	1	1.466			
	Lat	1	0.769			
OCG				0.553	553	94
	LAO	1	0.219			
	RPO	1	0.127			
	Right Lat	1	0.100			
	Right Lat cross table (AP)	1	0.107			

† IVP: Intravenous Pyelography, OCG: Oral Cholecystography.

* AP: anterior-posterior, LPO: left posterior-anterior oblique, RPO: right posterior-anterior oblique, Lat: lateral, LAO: left anterior-posterior oblique, Right Lat cross table (AP): The patient sleeps in a right-lateral situation and beam is horizontally projected

## Discussion

In this study, the fetal doses and consequence risks of the small head size and childhood cancer caused by radiation were obtained for different X-ray examinations, which these risks were not presented in previous studies. The calculated fetal doses were not more than 6 mGy for different radiographies; therefore, according to the literature, the obtained doses do not lead to abnormalities such as prenatal death, growth retardation, severe mental retardation, reduction of IQ, and organ malformation (2). However, risks of the small head size and childhood cancer for the radiographs of the abdomen, lumbar spine, pelvis, IVP, Cystography, and OCG were ponderable while they were negligible for radiographies of the skull, sinuses, and chest.

The new version of the computer program PCXMC was used in this study for fetal dose calculation which was not used in previous studies, although it was used in the other study for different purpose (14). Also an older version had been used in the study conducted by of Helmrot *et al*, but the version in this study was more developed because the new version had advantages such as using the new tissue weighting factors introduced in ICRP Publication 103 (9, 10). In this study, the calculated uterus dose was assumed to be equivalent to fetal dose (Fetus age <12 weeks). This assumption was also presumed in other studies (9, 11). 

In addition a necessary word of caution is that the fetus is more sensitive to radiation effects during the first weeks of pregnancy (12). In the present study, fetal dose was obtained for abdomen radiography in views of AP and PA as 1.22 and 0.60 mGy, respectively. However, in study of Helmrot *et al*, fetal dose was obtained for this radiography at view of AP as 0.92 and in study of McCollough *et al* for patients with thicknesses of 21 and 33 cm as 1 and 3 mGy, respectively (9, 13). Fetal dose was calculated for lumbar radiography in views of AP and lateral as 1.62 mGy, while in studies conducted by Helmrot *et al*, and McCollough *et al*, were obtained as 1 and 1.49 mGy, respectively. 

The received dose by fetus in frontal and lateral views of the chest radiography was 0.001 mGy, similar to study of Helmrot *et al*, but it is different from 0.002 mGy obtained in McCollough *et al* study (9, 13). For the pelvis radiograph in AP and PA view, fetal doses were calculated as 1.23 and 0.59 mGy, respectively, while in the Helmrot *et al* study, it was reported as 1.06 mGy for the AP view (9). As shown in table II, fetal doses were calculated in frontal position of the abdomen, lumbar spine, and pelvis radiographies in current view of AP and also in the uncommon PA view and it was found that the fetal dose would strongly be reduced in PA views comparing with AP views, and definite reason for the mentioned fact is a remarkable reduce in radiation intensity in PA views by the bones of the pelvis and spine before reaching to the fetus. 

In the present study, the fetus doses were calculated in lumbar spine radiography for seven different views, while in work conducted by the Helmrot *et al*, they had only been investigated in two views as AP and Lateral, and in McCollough *et al* study the fetal dose had not been presented separately (9, 13). The obtained realities can be used for considerable reduction of the fetus dose in emergency radiographs of the abdomen, lumbar spine and pelvis for pregnant women. In this study, fetal doses for some special examinations were also obtained and shown in table III. For radiography of IVP, the total dose received by the fetus (4.72 mGy) in the current study was in close agreement with the reported values in study of Helmrot *et al* (4.6 mGy), and in Osei *et al* study (4.8 mGy) (9, 11). Also in OCG examination, there was a relative agreement between total dose received by the fetus (0.55 mGy) in this work and value presented in the study of Osei *et al* (0.6 mGy) (11). 

But for radiography of bladder, the higher fetal dose (5.81mGy) was found in the current work compared to Osei *et al* survey (3.9 mGy) that can be attributed to differences in techniques and number of views investigated in the current work compared to Osei *et al* survey (11). In the present study, fetal dose values corresponding to different views were separately shown while these values were totally presented by Helmrot *et al* and Osei *et al* and not in detail (9, 11). The fetus dose values shown in tables II and III were obtained for an ordinary adult patient and field sizes were set according to reference guides. Obviously, they would be different if the patient be thinner or fatter. Also, if the field sizes are bigger or smaller than standards; subsequently, fetal doses would be increased or decreased. 

Consequently, findings of this study showed that the resulting fetal dose from radiographs of chest, skull, and sinuses were almost zero and do not lead to any abnormalities in the fetus, but the risks of childhood cancer and small head size due to radiographies of abdomen, lumbar spine, and pelvis areas were ponderable. In this study, the fetal dose variations in different views were also provided for some common radiography and some special examinations separately. The results of this study can be beneficial in avoiding of unnecessary termination of pregnancy as a result of fears about the wellbeing of the fetus after radiation exposure. Also, these findings can be helpful in decisions making regarding the pregnant women radiographies management in a way that the fetus would be received the possible lowest dose.
